# Adjuvant therapies for resected limited-stage small cell lung cancer

**DOI:** 10.1016/j.xjon.2021.05.018

**Published:** 2021-06-17

**Authors:** Savvas Lampridis

**Affiliations:** Department of Thoracic Surgery, 424 General Military Hospital, Thessaloniki, Greece

To the Editor:

**Figure:**
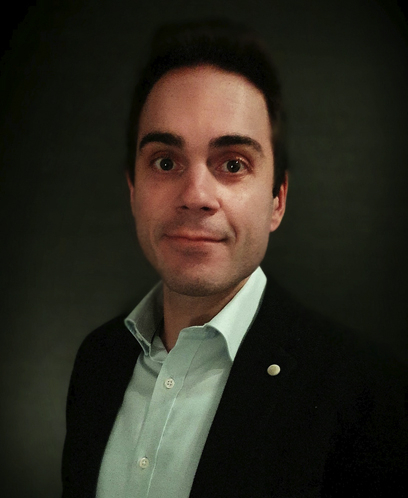

The author reported no conflicts of interest.

The *Journal* policy requires editors and reviewers to disclose conflicts of interest and to decline handling or reviewing manuscripts for which they may have a conflict of interest. The editors and reviewers of this article have no conflicts of interest.

It is estimated that less than 5% of patients with small cell lung cancer (SCLC) are amenable to surgical resection.^1^ As a result, the role of adjuvant therapies in resected SCLC has not been extensively investigated thus far. It is within this context that I read with great interest the article by Zhou and colleagues^2^ regarding predictors of survival following resection of limited-stage SCLC. The authors demonstrated a survival benefit from adjuvant chemotherapy but no significant advantage from postoperative radiotherapy (PORT) or prophylactic cranial irradiation.

In the staging system introduced by the Veterans Administration Lung Cancer Study Group, limited SCLC was originally defined as tumor volume that could be treated within a tolerable single radiotherapy port.^3^ Historically, however, the categories of limited and extensive disease have been inconsistently defined and used.^4^ Currently, it is recommended that only patients with clinical stage I-IIA (T1-2 N0 M0) SCLC and negative pathologic mediastinal staging are candidates for surgery as initial treatment.^1^ In the study by Zhou and colleagues,^2^ 11% of patients had T3 or T4 tumors, and 38% had N1 or N2 disease. Although several reasons may exist for patients with locally advanced SCLC to undergo surgery, more information on these reasons is important to draw clinically meaningful conclusions. For instance, unsuspected N2 involvement at surgical resection differs significantly from preoperatively identified N2 disease. Retrospectively derived results from the former subgroup of patients do not necessarily apply to the latter. Particularly regarding N2 involvement, it should be noted that 83% of patients had positron emission tomography–computed tomography but only 63% underwent mediastinoscopy or endobronchial ultrasonography. The underuse of mediastinal staging procedures makes the interpretation of the results even more challenging.

Interestingly, Zhou and colleagues^2^ found that PORT did not have a significant effect on overall survival for pN0 or pN-positive, limited-stage SCLC. The limitations of the study are well explained by the authors, who recommend decisions regarding the use of PORT to be made on an individual basis. It could be added here that the extent of nodal assessment performed at surgery should be taken into consideration in such decisions. The authors also compare the impact of PORT on survival with similar results observed in non–small cell lung cancer; however, such a comparison may be misleading because of the more aggressive biologic behavior exhibited by SCLC. Furthermore, the authors have failed to discuss a review of 3017 patients with limited-stage SCLC enrolled in the National Cancer Database indicating that PORT was associated with a 5-year overall survival benefit among those with pN2 disease (29% vs 18.6%; *P* < .001).^5^ In the same study, there were no differences in survival among patients with pN1 stage, whereas those with pN0 disease had decreased 5-year overall survival with PORT (39.3% vs 46.3%; *P* = .07). Stratification of patients according to specific pN stage in the study by Zhou and colleagues^2^ could provide more useful insights into the use of PORT.

The authors are to be congratulated for analyzing a relatively large cohort of patients undergoing surgery for SCLC, and their study contributes to the existing body of evidence on the surgical management of this disease. Prospective trials are anticipated to answer many of the remaining questions regarding the role of adjuvant therapies in resected SCLC.
